# Minimally Invasive Nasal Sampling in Children Offers Accurate Pneumococcal Colonization Detection

**DOI:** 10.1097/INF.0000000000002454

**Published:** 2019-10-01

**Authors:** Elissavet Nikolaou, Annie Blizard, Sherin Pojar, Elena Mitsi, Esther L. German, Jesús Reiné, Helen Hill, Paul S. McNamara, Andrea M. Collins, Daniela M. Ferreira, Simon P Jochems

**Affiliations:** From the * Department of Clinical Sciences, Liverpool School of Tropical Medicine, Liverpool, United Kingdom.; † Department of Respiratory Medicine, Royal Liverpool University Hospital, Liverpool, United Kingdom; ‡ Department of Child Health, Alder Hey Children’s NHS Foundation Trust Hospital, Liverpool, United Kingdom; § Department of Parasitology, Leiden University Medical Center, Leiden, Netherlands

**Keywords:** pneumococcus, children, synthetic absorptive matrices, colonization, quantitative PCR

## Abstract

Nasopharyngeal colonization of potential respiratory pathogens such as *Streptococcus pneumoniae* is the major source of transmission and precursor of invasive disease. Swabbing deeply the nasopharynx, which is currently recommended by World Health Organization, provides accurate pneumococcal detection but is unpleasant. We showed that nasal lining fluid filter strips offer equal detection sensitivity.

*Streptococcus pneumoniae* (Spn, pneumococcus), which naturally inhabits the nasopharynx of 40%–95% of infants without causing disease,^1^ is one of the most frequent causes of bacterial infection in children. This bacterium accounts for about 38% of childhood deaths caused by pneumonia,^2^ which is the leading cause of death in children <5 years worldwide.^3^ Therefore, detection of pneumococcal colonization is of a great importance, as it is the primary reservoir for transmission and prerequisite of invasive disease.

There are a variety of sampling techniques for detecting nasopharyngeal colonization with different detection sensitivities. In adults, nasopharyngeal swab (NPS) and nasopharyngeal wash cultures have been shown to detect higher rates of *S. pneumoniae* colonization than oropharyngeal swabs.^4^ However, sampling in children is challenging as swabs and aspirates can cause significant discomfort. Saliva sampling, which is painless to collect, has been successfully used to detect pneumococcus in children instead of NPS or oropharyngeal swabs; however, due to its polymicrobial nature might give false-positive results when using molecular methods.^5^

On the other hand, sampling of nasal lining fluid using synthetic absorptive matrices (SAM) does not cause discomfort and has been used to detect respiratory syncytial virus infection in a pediatric intensive care unit setting.^6^ Whether such minimally invasive samples could detect bacteria, including pneumococcus, has not been assessed yet, and there is a lack of evidence on whether nasal sampling is as sensitive as nasopharyngeal sampling for detection of carriage. The World Health Organization thus recommends NPS for pneumococcal colonization detection in children and both NPS and oropharyngeal swabs in adults.^7^

Recently, limitations of detection in conventional microbiology have led to the increased employment of PCR-based methods. The latter detects pneumococcus at low densities and thus offers high sensitivity for colonization detection. For detecting pneumococcal DNA in clinical samples, World Health Organization recommends the use of quantitative PCR (qPCR) targeting the well-conserved autolysin-encoding gene *lytA*.^7^

The present study aimed to test whether SAM can be used to accurately assess pneumococcal colonization by comparing the sensitivity (colonization rates and density) for detecting pneumococcal colonization in children between SAM and NPS using l*ytA* qPCR. We also compared the results obtained with NPS cultures.

## MATERIALS AND METHODS

### Study Design and Ethics Statement

SAM (Nasosorption, Hunt Developments) and NPS (Transwab, Sigma) samples were collected from 49 children of 1–5 years of age who were under general anesthesia for unrelated reasons. Samples were collected after onset of general anesthesia but prior to start of their planned procedure (dental extraction, magnetic resonance imaging, orthopedic, or plastic surgery). SAM samples were collected first, to prevent contamination of the anterior nares following withdrawal of the NPS, by inserting the SAM strip into the nostril and keeping it in touch with the mucosal surface for 1 minute. To assess pneumococcal colonization, NPS samples were placed in 1 mL skim milk, tryptone, glucose, and glycerin medium, 100 µL of which was cultured on Columbia blood agar supplemented with 5% horse blood (PB0122A, Oxoid/Thermo Scientific), and 80 μL gentamicin 1 mg/mL (G1264-250 mg, Sigma-Aldrich Co Ltd). Plates were incubated overnight at 37^o^C and 5% CO_2_. Pneumococcal serotype was confirmed by latex agglutination (Statens Serum Institute, Copenhagen, Denmark). SAM samples and the residual NPS samples were frozen at −80^o^C to be used for DNA extraction and qPCR.

Informed consent was obtained from the parents of all children after a thorough explanation of the study. This trial was approved by The National Health Service Research and Ethics Committee (17/NW/0663) and was sponsored by the Liverpool School of Tropical Medicine. All experiments were adapted to the relevant regulatory standards (Human Tissue Act, 2004).

### Pneumococcal DNA Extraction from SAM and NPS Samples

On the day of the extraction, SAM samples were thawed for 30 minutes on ice. Hundred microliters of Luminex assay diluent (Thermofisher, Basingstoke, UK) filter, which was then 1503*g* (4000 rpm) for 10 minutes at 4^o^C. After centrifugation, the eluted liquid was moved to a clean Eppendorf tube and centrifuged at 16,000*g* for 10 minutes at 4^o^C. The supernatant was removed, and the pellets were used for DNA extraction. DNA extraction was performed using the Agowa Mag mini DNA extraction kit (LGC genomics, Berlin, Germany) and manufacturer’s instructions were followed. For NPS samples, 200 µL raw material was defrosted, and DNA was extracted using the same procedure.

### Quantification of Pneumococcal DNA in SAM and NPS Samples by lytA qPCR

Colonization density in both SAM and NPS samples was determined by qPCR targeting the *lytA* gene (10) using the Mx3005P system (Agilent Technologies, Cheadle, UK). The sequences of the primers and probes used are: *lytA* forward primer: 5′-ACG-CAA-TCT-AGC-AGA-TGA-AGC-A-3′; *lytA* reverse primer 5′-TCG-TGC-GTT-TTA-ATT-CCA-GCT-3′; *lytA* probe: 5′-(FAM)-TGC-CGA-AAA-CGC-TTG-ATA-CAG-GGA-G-(BHQ-1)-3′. For the standard curve, pneumococcal DNA was extracted using the QIAamp DNA mini kit (Qiagen, Hilden, Germany). Samples were considered positive if 2 or all triplicates yielded a C_T_ < 40 cycles. Multiple experiment analysis was performed, and cross experiment threshold was calculated by using interrun calibrators.

### Statistical Analysis

Statistical analysis was performed by GraphPad Prism version 5.0 (California). Data were log-transformed where appropriate. To distinguish between parametric and nonparametric data a Kolmogorov-Smirnoff normality test was performed. To quantify association between groups, the Pearson correlation test was used for parametric groups. Densities between methods were compared using paired nonparametric Wilcoxon tests. Sensitivity was compared using χ^2^ tests. Differences were considered significant if *P* ≤ 0.05. Sensitivity between culture and molecular methods were analyzed 1-sided as molecular methods are more sensitive, all other tests were performed 2-sided.

## RESULTS

### Both SAM and NPS qPCR Detect Equal Pneumococcal Colonization Rates Higher Than NPS Cultured Samples

**FIGURE 1.**
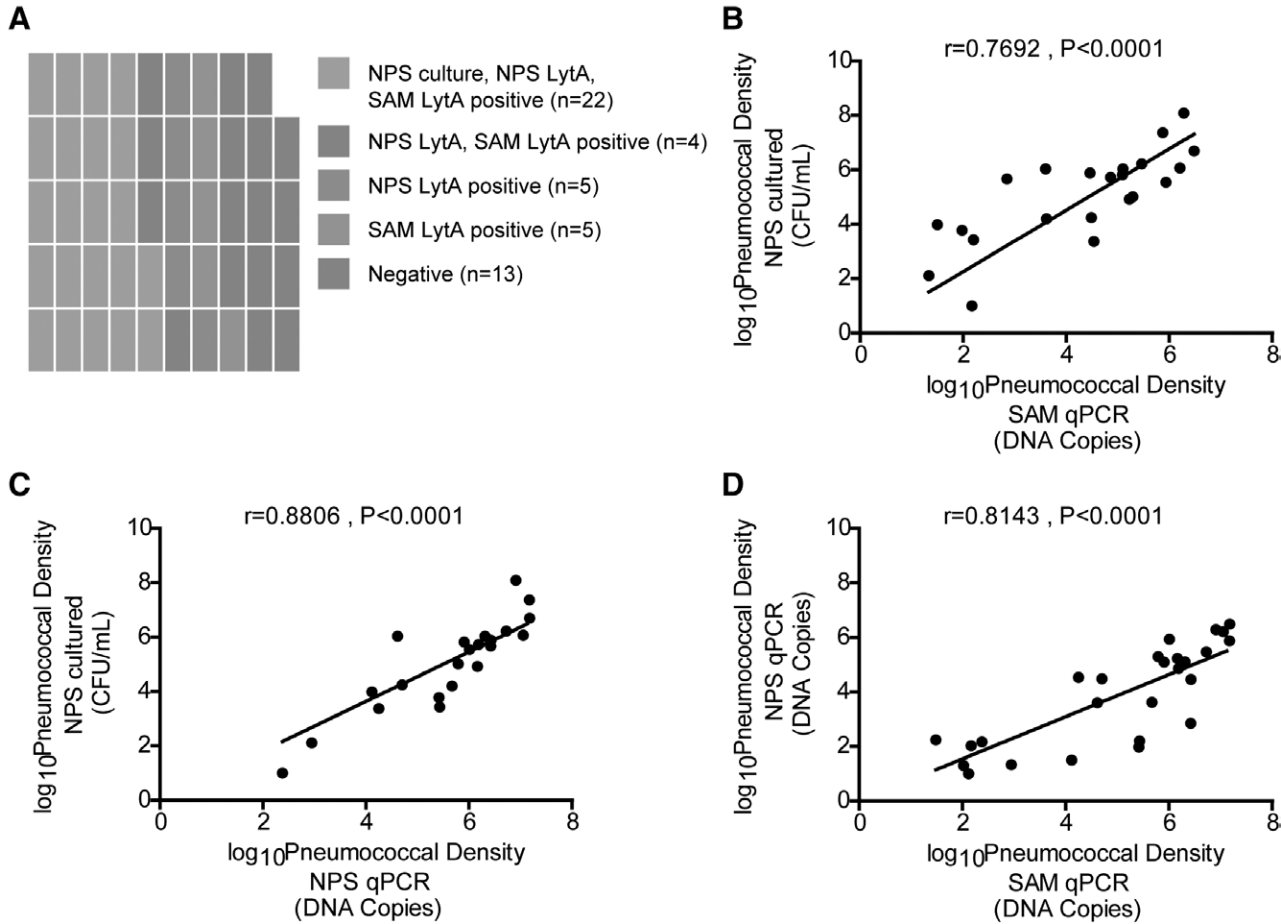
Correlation of colonization presence and densities between detection methods. A. Spn presence by all methods. Waffle diagram showing numbers of children positive for Spn by any combination of the 3 compared methods. Every square represents 1 individual with color indicating identification. B. Density of Spn in SAM qPCR versus NPS cultured. C. NPS qPCR versus NPS cultured. D. SAM qPCR versus NPS qPCR. Points represent children positive for Spn and a linear regression line is added. Data were log transformed. A. Pearson test was used to measure correlation between the methods of pneumococcal detection. B. Twenty-two children were positive for Spn by both SAM qPCR and NPS cultured (*r* = 0.7692). C. Twenty-two children were positive for Spn by both NPS qPCR and NPS cultured (*r* = 0.8806). D. Twenty-six children were positive for Spn by both SAM qPCR and NPS cultured (*r* = 0.8143).

SAM and NPS samples were collected from 49 of 50 children enrolled in the study and used in this analysis. Using culture of NPS, 22 of 49 (44.90%) children were positive for Spn (Fig. 1A). Serotypes/groups identified were SPN15 (7), SPN23 (4), SPN non-vaccine type group G (3), SPN11 (2), SPN19 (2), SPN3, SPN10, SPN non-vaccine type group E, and SPN non-vaccine type group I. All culture-positive children were also positive by molecular detection from NPS and SAM. Another 4 children were positive for Spn by *lytA* qPCR from both SAM and NPS (Fig. 1A). Moreover, another 10 children were positive by lytA qPCR either in SAM or NPS samples (5 each) (Fig. 1A). Thus, NPS and SAM *lytA* qPCR each detected 31 of 49 (63.27%) children positive for Spn (*P* = 1.0) and agreed in 39 of 49 (79.6%) of them. In total, the number of positive colonized children detected by qPCR in both SAM and NPS samples was 1.4-fold higher than those detected in NPS cultured samples (*P* = 0.034).

### Pneumococcal Colonization Densities Measured by all Three Methods Correlate Significantly to Each Other

There was a significant correlation between bacterial load determined by SAM *lytA* qPCR, NPS *lytA* qPCR, and NPS cultured (*P* < 0.0001, Fig. 1). In the majority of cases, pneumococcal densities measured by NPS qPCR were higher than those detected by NPS cultured (19/22, 86.36%, *P* < 0.0001). Four samples were positive by both SAM and NPS qPCR but not NPS cultured, with densities ranging between 10-176 DNA copies from SAM and 31-149 DNA copies from NPS. Another 5 samples were positive only by SAM *lytA* qPCR with densities 10-151 DNA copies. Another 5 were positive only by NPS *lytA* qPCR with densities 60-205 Spn DNA copies. Pneumococcal densities calculated by NPS qPCR were usually higher than those detected by SAM qPCR in children positive in both samples (24/26, 92.31%).

## DISCUSSION

Our results showed that SAM can be used as an alternative method to the current gold standard NPS^8^ for pneumococcal detection in children with equal detection sensitivity. NPS sampling is associated with substantial discomfort.^4^ SAM sampling targeting the anterior nares is a less invasive technique than NPS sampling, where a sample is collected from the nasopharynx. We have previously demonstrated that SAM sampling has low levels of discomfort, pain, and lacrimation in adults.^8^

The number of volunteers that were identified as Spn colonized by *lytA* qPCR (colonization rate) was higher than the number found by classical culture, as was expected. SAM qPCR detected equal numbers of Spn-positive children as NPS qPCR (31/49, 63.27%) and agreed in 39 of 49 (79.6%) of cases, demonstrating that SAM sampling is a sensitive and specific alternative to NPS for pneumococcal detection in children. The children that were identified as carriers from only NPS or SAM were predominantly low-density colonized and the discrepancy between the 2 sites might thus be stochastic. However, it is not impossible that differences between the 2 sites (anterior part of the nose and nasopharynx) exist in terms of microbiota composition. Additionally, we observed that pneumococcal densities in Spn-positive volunteers detected by NPS qPCR are higher than those detected by SAM qPCR although this did not lead to differences in numbers of identified carriers. It is possible that swabbing collects more sample than absorption by SAM, although this did not affect sensitivity of Spn detection.

As we did not perform molecular serotyping in this study, we cannot be certain that the same serotypes were picked up between the 2 sites. However, the good concordance with culture results and correlation between densities at different sites suggest that the same pneumococcal reservoir was sampled by SAM and NPS.

Previously, using the Experimental Human Pneumococcal Challenge model of infection in which healthy adults were challenged with type 6B pneumococcus, detection of pneumococcus in the nose of adults using SAM once Spn colonization was established was low.^9^ At day 2 and 6 after 6B exposure, only 1 of 9 (11.1%) and 1 of 7 (14%) Spn-positive adults (carriers by classical culture of nasal washes) was found to be Spn positive by SAM qPCR. Possible explanations for this discrepancy are differences in anatomy, physiology, and nasal/oral microbiome between both groups and the possible change of colonization niche from the nasopharynx to oropharynx in adults.^5^ Our findings are in agreement with decreasing sensitivity of nasal swabs, when compared with NPSs, to detect pneumococcal carriage in adults compared with children.^10^ The increased presence of pneumococcus in the anterior parts of the nose in children compared with adults could offer an explanation as to why children are transmitting more than adults.

In conclusion, our findings support that SAM sampling is a robust method for accurate detection of pneumococcus in children that could be employed during clinical trials and large epidemiologic studies.

## ACKNOWLEDGMENTS

The authors like to thank all children for their participation and their parents for providing informed consent. Also, they like to thank the Liverpool Alder Hey Children Hospital for supporting this research.
